# A Stitch in Time Saves Nine: Predicting Internet Addiction Levels of Preservice Teachers

**DOI:** 10.1007/s11126-025-10120-2

**Published:** 2025-02-17

**Authors:** İsmail Şan, H. Gülhan Orhan Karsak, Curtis J. Bonk, Derya Karadeniz

**Affiliations:** 1 Department of Educational Sciences, Faculty of Education, İnönü University, 44280 Malatya, Turkey; 2 Faculty of Arts and Science, Department of Educational Sciences, Kırklareli University, 39000 Kırklareli, Turkey; 3 Instructional Systems Technology Department, School of Education, Indiana University, Bloomington, IN USA

**Keywords:** Internet Addiction, Emotion Regulation Skills, Extracurricular Study Habits, Preservice Teachers

## Abstract

The present study aimed to explore the potential moderating effects of extracurricular study habits, internet usage duration, gender and emotion regulation skills on internet addiction of preservice teachers. The sample consisted of 492 preservice teachers (308 female) from 10 different institutions in Turkey, who voluntarily provided data. The findings revealed that emotion regulation skills, particularly reappraisal, interacted with daily internet usage time in predicting internet addiction. High levels of reappraisal emotion regulation were linked to lower levels of internet addiction, regardless of the duration of internet use. Conversely, for individuals with lower reappraisal abilities, a positive correlation emerged between internet usage time and internet addiction. Furthermore, extracurricular study habits significantly contributed to the prediction of internet addiction in a positive way, as evidenced by both regression and correlational analyses. These findings underscore the importance of considering emotion regulation alongside study habits and other physiological factors to better understand and address internet addiction in preservice teachers. The implications of the findings for educational policy and teacher education are discussed.

## Introduction

The term 'internet addiction' describes a condition marked by an excessive and uncontrollable reliance on the internet, frequently involving the use of social media, gaming platforms, online shopping, and a multitude of other digital activities [92, 156, 160]. Individuals may turn to the internet as a source of stress relief, to occupy idle time, or to fulfill their social needs,nevertheless, its ever-present nature can foster a dependency [23, 41, 80, 97]. Those with deficiencies in self-regulation, social isolation or psychological distress are particularly vulnerable to the development of problematic internet use [9, 49, 84, 154]. The manifestation of addiction is typically characterized by a pervading need to remain connected and a sense of unease when not online, which can have detrimental effects on professional, educational and social activities [11, 64]. While the phenomenon of internet addiction can affect any age group, it is particularly prevalent among adolescents and young adults [4, 30, 143]. Such addiction is especially true in instances when these individuals are experiencing feelings of isolation or personal difficulties [24, 152]. In this study, we aimed to examine the individual factors contributing to internet addiction of pre-service teachers.

In this study, firstly, variables such as extracurricular study habits and emotion regulation skills will be emphasised in order to address the individual factors affecting pre-service teachers' internet addiction in a more comprehensive framework. Then, the method section will be presented in detail in order to examine the interaction of the participants' demographic characteristics (such as gender and daily internet usage time) with these variables; the scales used here and the data collection process will be explained. In the next stage, the findings will be evaluated through descriptive statistics as well as multiple regression and moderating effect analyses. In line with the results obtained, the predictive role of both extracurricular study habits and emotion regulation skills on internet addiction will be interpreted and the findings will be related to the literature. At the end of the study, suggestions for the development of preventive strategies for internet addiction in educational environments and future research perspectives will be discussed.

### Extracurricular Study Habits and Internet Addiction

The development and adjustment of individuals are seen as being particularly dependent on their study habits [34, 56, 89, 99, 119]. Poor study habits have been identified as a source of stress in both work and educational contexts [5, 13, 26, 29, 54]. This kind of study habit is associated with a range of problematic behaviors, including alcohol, drug and other substance abuse [18, 46, 59, 106, 120, 126, 144].

In the past few years, researchers have investigated the link between poor study habits and a new phenomenon: Web addiction. The findings of such studies have revealed that students exhibiting poor study habits are more likely to exhibit high levels of internet addiction [28, 148, 153].

While deficient study habits have been demonstrated to presage a plethora of maladaptive consequences, including the development of internet addiction [23, 96], it is important to recognize that individuals do not all exhibit the same degree of vulnerability in the context of either adverse or beneficial environments [93, 121, 164]. The Differential Susceptibility to Environmental Influences Theory (DSEIT [12],) proposes that individuals demonstrate disparate degrees of sensitivity to stressful circumstances or unfavorable environmental conditions, contingent upon their vulnerability level. Factors that contribute to this vulnerability encompass genetic predispositions (e.g., serotonin transporter gene polymorphisms), negative emotionality (e.g., anxiety and depressive tendencies), and psychosocial characteristics (e.g., low self-regulation or impulsivity).

Although the topic is not extensively covered in the prevailing academic literature, evidence suggests a positive correlation between extracurricular study habits (ESH) and teacher behaviors, as well as peer support. These findings have been reported in studies by Lamb [95] and Lai et al. [94]. In Turkey, teachers are expected to enhance their pedagogical skills with a view to imparting extracurricular study habits within their students as well as many other competence areas [103]. Therefore, it is reasonable to hypothesize that poor study habits may increase the risk of developing internet addiction, particularly among individuals who are particularly vulnerable to such a development.

### Emotion Regulation and Internet Addiction

With respect to individual attributes, there is a growing body of evidence suggesting that self-regulation may play a significant contributing role in addictive behaviors, including internet addiction. This assertion is supported by findings from several prominent studies, including those conducted by Baumeister [8], Oh [108], Setyawan and Masykur [124], and Zhu [163]. Those who exhibit deficits in self-regulation may be more prone to engaging in addictive behaviors as they encounter obstacles in their capacity to monitor, evaluate, and regulate their online activities [25, 41, 97, 105]. Emotion regulation (ER), defined as the variability in emotional responses, has been identified as a crucial factor influencing an individual's capacity for emotional self-regulation, as reported by the subject (e.g., [68, 104, 139, 158]).

The ER-R (Reappraisal) and ER-S (Suppression) subscales are two frequently employed measures within the context of emotional regulation. It is thought that ER-S reflects an individual’s ability to suppress emotions under normal circumstances; thus, often signaling an underlying negative outlook. In contrast, ER-R reflects an individual's ability to adapt their focus according to changing circumstances [66, 68]. In the presence of some moderately challenging tasks to be completed during daily activities, ER-S is often associated with individuals disregarding their own emotions in order to adapt to environmental demands. This situation can result in a greater experience of stress, or the development of a stronger expectation that the same level of challenge will be present in the future, if the task is ongoing [113, 115, 134].

Studies have revealed that there are many factors related to emotion regulation [135]. Empirical evidence indicates that higher scores on the ER-R subscale are associated with several aspects of positive psychological functioning, including the ability to regulate emotions effectively, enhanced social competence, and a greater tendency to engage in prosocial behaviors. These observations have been documented in studies conducted by a wide array of researchers (e.g., [19, 20, 58, 63, 70, 79, 141, 149]). By contrast, excessive ER-S has been linked with psychopathology and a range of other maladaptive behaviors and indicators, including emotional disturbance, externalizing problems and internalizing disorders [3, 14, 50, 55, 61, 69, 81, 90].

The available research on the relationship between emotional regulation (ER) and internet addiction, and its associated symptoms, has yielded inconclusive results. This is evidenced by the varying findings reported in the literature [31, 111],Şahin & Korkmaz, 2021; [151]. To illustrate, Busetta et al. [21] and Khodadadifard et al. [82] demonstrated that schoolchildren exhibiting symptoms of internet addiction exhibited significantly diminished levels of perceived stress compared to those without internet addiction. In other studies, LaRose et al. [97] and Zhang et al. [160] discovered that problematic internet usage was linked to ER-S,yet, the direction of this relationship varied contingent on the context of the task [61]. Specifically, the association between problematic internet usage and ER-S was stronger for cognitive control tasks compared to social interaction tasks [25, 27, 77, 83, 142].

In a recent study, Liang et al. [98] observed a reduction in emotional regulation (ER) in individuals identified as being at high risk of developing internet addiction, compared to those deemed to be at lower risk. This reduction in ER was evidenced both before and after the induction of positive and negative emotions. One might preliminarily conclude from these results that measures of emotional regulation may predict the development of internet addiction. However, the context in which these measures are administered appears to influence the results [25, 67, 80, 147, 155, 156].

### The Combined Effect of ESH and ER on Internet Addiction

As pointed out in Goleman [62] a growing body of literature has recently emerged concerning the moderating effect of emotion regulation in the relations of emotional intelligence (EI) factors (e.g., self-awareness, self-regulation, empathy, social skills, motivation) in terms of individuals' socio-emotional adaptation. Nevertheless, the observed patterns of interaction exhibit certain degrees of variability (see [40, 109, 110, 122, 159]). For instance, in adolescents aged 12 to 18 years, the capacity to regulate one's emotions was found to be an encouraging indicator for the utilization of constructive coping strategies in response to academic pressure. Nevertheless, this was solely observable in individuals who demonstrated a moderate to high level of emotional regulation [68, 110].

In individuals aged between 18 and 25 years, the development of empathy skills was associated with a stronger interpersonal relationship and a lower risk of substance misuse, particularly in those who had developed advanced reappraisal strategies [122, 159]. Among younger adolescents (ages 10–15), high self-awareness was associated with a reduced likelihood of engaging in risky behaviors, such as alcohol use. This association was limited to adolescents who demonstrated developed suppression skills as a form of ER [140]. Additionally, researchers such as Boekaerts and Corno [15], Compass et al. [38], Duckworth and Seligman [45], Evans and Kim [52], MacCann et al. [100], and Skinner and Pitzer [127] have reported that higher ESH is associated with greater success in managing internalizing problems among children with low ER during periods of stress. Therefore, relatively high ER-R and ESH appear to function as protective factors, potentially mitigating the adverse impact of stress sources on individual outcomes (e.g., [53, 57, 67, 101, 125, 128, 150]).

### Gender and Daily Internet Usage Time as Moderator

The recognition of significant discrepancies between the genders in a multitude of areas, including biological, physical, cognitive, and social-emotional development, has given rise to an increasing number of researchers advocating for the integration of gender-related considerations into the study of psychopathology [2, 107, 137, 157]. Recent research provides evidence for gender differences in relations between extracurricular study habits, emotion regulation and individual outcomes (e.g., [32, 43]. To illustrate, Rueckert et al. [117] and Spinrad and Eisenberg [131] discovered that elevated general emotional responsiveness was predictive of higher levels of empathic concern in adolescent girls with high levels of ESH, yet not in boys of a similar age. As previously outlined by the MoNE [103], the influence of an external environment on an individual's psychological and behavioral development is not uniform. It can be observed that not all individuals are equally affected by environments that are either conducive to positive growth or detrimental to it. This concept has been further reinforced by research conducted by Hyde [78] and Else-Quest et al. [48]. These findings have emphasized the necessity to examine gender as a potential moderator when attempting to predict psychological disorders from biosocial characteristics.

In a considerable number of studies examining internet addiction, the extent of excessive online usage has been identified as a crucial factor in determining the presence or absence of internet addiction. Young [156] observed that individuals with Internet addiction spent, on average, 39 h per week online, whereas individuals without such a disorder spent a mere 5 h online per week. In a similar vein, Chen and Chou [33] observed that the high-risk group averaged about 20 h per week online, whereas the low-risk group averaged just 9 h. In the study conducted by Chou and Hsiao [36], it was observed that individuals with addictive tendencies spent approximately 20 to 25 h per week online, whereas individuals without such tendencies spent approximately one-third of that time online.

In these studies, it was also found that individuals with addictive behaviors demonstrated a preference for two-way communication functions, such as chat rooms, multi-user games, newsgroups, and email. In contrast, individuals without addictive behaviors exhibited a preference for one-way functions, such as information search and Web browsing. Young [156] posited that addictive behavior is contingent upon the specific applications utilized, rather than the general use of the internet. This finding was corroborated by other researchers, including Chou et al. [35] and Chou and Hsiao [36] have demonstrated that, within the context of Asian culture, individuals struggling with addiction tended to utilize electronic Bulletin Board Systems (BBS) and email functions with greater frequency. In studies conducted with the Turkish sample, internet addiction has been found to be associated with specific applications, including social media, online games, and video streaming platforms [42]. Social media applications (e.g., Instagram, Twitter, Facebook, etc.) have been identified as the primary addictive factors, while online multiplayer games (e.g., PUBG, League of Legends) have also been highlighted as a significant risk factor [6]. It is evident that these applications offer considerable socialization potential and an element of competition, which together result in users spending considerable time on the applications, and exhibiting signs of addiction [65]. Concomitantly, video streaming platforms—such as YouTube and Netflix—have been identified as another factor contributing to addictive use [72]. Empirical studies on internet addiction in Turkey have revealed that these applications are used extensively, particularly among the young population, and that they exert negative effects on academic, social, and occupational functioning over time [30]. In this context, the researchers concluded that studies examining the relationship between internet addiction and extracurricular study habits and emotion regulation skills among education students are limited and warrant further research. Therefore, we explored whether the hypothesised relationships varied according to the gender and DIUT of the individuals concerned.

### The Present Study

The object of the present investigation was to examine the potential moderating roles of extracurricular study habits (ESH) and emotion regulation skills (ER) on internet addiction (IA). It was hypothesized that internet addiction would be more prevalent when both ESH and ER were low. Additionally, the study explored the potential for gender to moderate these relationships, although the specific nature of this potential moderation was not defined prior to the commencement of the research.

## Methods

### Participants

In the study, predictive design was used in the context of quantitative research method. The study cohort comprised 492 pre-service teachers (PSTs), aged 18–22 (308 female, 492 medium-income from 10 universities across Türkiye. Of these 492 preservice teachers 225 were living with their parents, 171 were freshmen, and 390 had an average perceived academic level of achievement. Notably, 365 were occasional absentees from classes. Importantly, the majority of these respondents (i.e., 245) primarily connected to the internet from home, whereas 484 utilize a mobile phone to access the internet. The mean time per day spent online by the participants was 7.51 h, with an average age of 20.6.

These individuals completed a survey package assessing ESHS, ERI, and IA. The data collection tools were completed online at the commencement of the lecture hours of the lecturers employed at faculties of education of 10 different universities in Turkey. The data was collected after the requisite information about the research had been provided to the participants. Participation in the study was voluntary, and this was confirmed by completion of the voluntary participant consent form. Importantly, approval for the study was obtained from the ethics committee.

### Data Collection Process

Once all participants had been made aware that their involvement in the study was entirely optional, they were invited to complete a comprehensive 60-item scale package, which they could complete in approximately 20 min. In the measurements where the subjective valuation approach was employed, the demographic variables were inquired of in a manner so as to prevent the disclosure of the individuals' identities. The study population consisted of those PSTs who volunteered to participate in the application on the day the scales were administered. The study was explained to the students who were present in the classroom, and their informed consent was obtained. The participants were informed by the researchers and the study implementers that they could request clarification regarding any aspects of the scales that were unclear to them.

Subsequently, each participant group was permitted approximately five minutes to peruse the items at a rapid pace. Once the participants had been provided with the requisite information, the data collection process commenced online. Subsequently, the participants were presented with the 14-item demographic information form, the 17-item five-point Likert-type extracurricular study habits scale, the 19-item five-point Likert-type internet addiction scale, and the 10-item seven-point Likert-type emotion regulation skills inventory. In addition, the participants were invited to provide a subjective evaluation of their internet addiction, extracurricular study habits and emotion regulation skills. The aforementioned scales were employed with the objective of determining the level of internet addiction, emotion regulation skills and extracurricular study habits exhibited by PSTs. All these data were collected in 2024.

### Measures

#### Internet Addiction Scale (IAS)

The scale developed by Hahn and Jerusalem [73] that was subsequently adapted into Turkish by Şahin and Korkmaz [136] was employed in the present study. That scale is comprised of 19 items, which have been grouped into three sub-dimensions: The scale comprises three sub-dimensions: Loss of Control (7 items), Desire to Stay Online More (4 items) and Negativity in Social Relationships (8 items). Each item was rated on a 5-point scale, ranging from 1 (never) to 5 (always), with higher scores indicating higher levels of internet addiction. The scores for each item were added together to create a total score for internet addiction. The Cronbach's alpha value for the overall scale was found to be 0.936, while the Cronbach's alpha coefficients were 0.873, 0.884 and 0.915 for the sub-dimensions, respectively.

#### Extracurricular Study Habits Scale (ESHS)

The extracurricular study habits of PSTs were evaluated using the After-School Study Habits Scale [51], a 17-item self-report scale designed to assess the strategy, motivation, environment, and planning components of these habits. The validity and reliability of this measurement tool had been previously established in the relevant literature. Participants indicated their responses on a 5-point Likert scale, ranging from 1 (not at all appropriate) to 5 (fully appropriate), with higher scores indicating higher levels of extracurricular study habits. After five items (items 7, 8, 9, 10, and 16) in the scale were found to be antithetical to the overall scale, they were reverse-coded in the calculations. A total score was then calculated in order to form a composite of extracurricular study habits. The data collected from this sample yielded a reliability coefficient of 0.769 for the entire scale. The respective values for the sub-dimensions are 0.839, 0.686, 0.663 and 0.873.

#### Emotion Regulation Skills Inventory (ERSI)

The Emotion Regulation Skill Inventory (ERSI), a 10-item self-report inventory developed by Gross and John [68] and adapted to the Turkish language by Eldeklioğlu and Eroğlu [47] was used for the measurement of emotion regulation skills. The validity and reliability of the ERSI were reported in Eldeklioğlu and Eroğlu [47], and it consisted of 'reappraisal' and 'suppression' dimensions, which do not yield meaningful results when collected together. Participants responded on a 7-point scale, ranging from 1 (strongly disagree) to 7 (strongly agree). Notably, those with higher scores on the Reappraisal dimension and lower scores on the Suppression dimension were considered to have higher emotion regulation skills. According to the data collected from this sample, the reliability coefficients were calculated to be 0.816 on the Reappraisal dimension and 0.721 on the Suppression dimension.

### Data Analysis

The initial analysis employed the use of measures of central tendency, such as the mean and median, as well as dispersion measures, such as standard deviation and interquartile range, in addition to frequency tables, in order to examine the raw data. This preliminary analysis enabled the investigation of the prevalence of internet addiction and extracurricular study habits, as well as emotion regulation abilities, among prospective teachers and the normality of these traits' distribution in accordance with demographic variables.

Subsequently, Pearson's correlation coefficient was calculated to examine the relationships between the study variables, in accordance with the guidelines set forth by Cohen et al. [37] for interpreting the magnitude of Pearson correlation coefficients. The objective of the third stage of the study was to employ hierarchical multiple regressions in order to examine the independent and interactive effects of gender and the daily internet usage time (DIUT), in addition to extracurricular activities and emotion regulation, on the phenomenon of Internet Addiction. Following the recommendations of Aiken and West [1], certain variables were converted into digital format using a process known as encoding. The variables included gender (coded male = 0, female = 1), place of residence (coded home = 0, dormitory = 1), and place of internet connection (coded home = 0, other = 1). To minimize multicollinearity, a mean score was calculated for each participant on the variables of academic achievement, attendance status, average DIUT, frequency of internet use for each purpose, extracurricular study habits, and emotion regulation skills prior to conducting the regression analyses. As proposed by Cohen et al. [37], to illustrate the significant interaction effects, three simple regression lines were constructed, one for low values, one for the average, and one for high values. In order to determine whether the slopes of the simple regression lines for ESH and ER differed from zero, post hoc evidence was employed [1, 76].

## Results

### Preliminary Analyses

Table 1 presents the descriptive statistics for the variables ER-R, ER-S, ESH, and IA as percentages. A sample size of 492 was used for all variables; no missing data was reported. The mean scores range from 65.8% (ER-S) to 69.0% (IA), indicating the average level of these constructs experienced by participants. The standard deviation for these variables varies from 12.8% (for ER-R) to 15.0% (for ESH), thus demonstrating the extent of variation in participants' responses.

**Table 1:** Descriptive statistics for all dependent variables

Variable	N	Mean	Median	Mode	SD	Skewness	Kurtosis	Min	Max
IA	492	69.0	68.0	54.0	14.9	0.1860	−0.615	34	100
ESH	492	67.7	68.0	73.0	15.0	0.0092	−0.615	32	100
ER-R	492	66.4	66.0	70.0	12.8	0.0634	0.110	33	100
ER-S	492	65.8	66.0	59.0	13.1	0.0215	0.281	29	100

The mean score for internet addiction (IA) was 69.0%, which suggests that participants exhibit moderate levels of internet addiction. The skewness value of 0.1860 indicated a slight positive skew, suggesting that while some participants report higher levels of internet addiction, the majority of scores were concentrated around the mean.

The mean score for extracurricular study habits (ESH) is 67.7%, which suggested that the participants engaged in these habits with a high level of intensity on average. The standard deviation of 15.0% indicated a moderate degree of variation among participants, reflecting differences in the consistency of engagement levels.

The results also indicate that participants demonstrated moderate engagement in emotion regulation skills, as measured by the ER-R and ER-S subscales. The mean score for reappraisal (ER-R) is 66.4%, while for suppression (ER-S) it is 65.8%. The slight positive skewness values (0.0634 for ER-R and 0.0215 for ER-S) imply a relatively uniform distribution, indicating that participants utilised these strategies in moderation with minor deviations.

Overall, the constructs under examination demonstrated moderate levels of intensity among the participants. The percentage values were typically concentrated between 65 and 70%. The observed variability in the constructs suggests that, while the majority of participants demonstrated these behaviors and skills at similar levels, there were notable individual differences, as evidenced by the standard deviation percentages. These findings emphasized the relative prevalence and intensity of each construct within the sample, indicating that both internet addiction and extracurricular study habits are frequently experienced, while emotion regulation strategies such as reappraisal and suppression were employed to a moderate and consistent extent.

The correlation matrix (Table 2) demonstrates a positive correlation between internet addiction (IA) and extracurricular study habits (ESH) (*r* = 0.600, *p* < 0.001), indicating a tendency for these variables to vary together among students. A negative correlation was identified between IA and Emotion Reappraisal (ER-R) (*r* = −0.149, *p <* 0.05), and a comparable negative correlation was observed between IA and Emotion Suppression (ER-S) (*r* = −0.273, *p* < 0.001). No statistically significant relationship was found between IA and the increase in (DIUT) (*r* = −0.059, *p* > 0.05). Furthermore, the correlation between IA and income level was determined to be weak but positive (r = 0.092, *p* < 0.05).

**Table 2:** Correlations for study variables

	1	2	3	4	5	6	7	8
1. IA	–							
2. ESH	0.600**							
3. ER-R	−0.149**	−0.362**	—					
4. ER-S	−0.275**	−0.267**	0.899**	—				
5. DIUT	−0.059	−0.249**	−0.054	−0.273**	—			
6. Income level	−0.005	−0.061	0.032	0.007	0.092*	—		
7. Class	−0.193**	0.086	−0.050	0.028	−0.020	0.011	—	
8. Academic Achievement	−0.048	0.064	−0.011	0.078	−0.038	0.076	0.115*	—
9. Attendance Status	0.054	0.030	−0.030	−0.066	0.031	−0.034	−0.066	−0.169**

*p < 0.01, **p < *0.001*

The correlations presented in Table 2 served to highlight patterns of association that proved useful in understanding behavioral trends associated with internet addiction.

### Regression Analysis

A hierarchical multiple regression was conducted to determine whether the inclusion of Extracurricular Study Habits (ESH) and subsequently Emotion Regulation skills (Reappraisal [ER-R] and Suppression [ER-S]) improved the prediction of Internet Addiction (IA) beyond the baseline variables of gender and DIUT. See Table 3 for full details on each regression model.

**Table 3:** Main and interactive effects of ESH, ER-R, ER-S, DIUT, and Gender predicting internet addiction levels

		Model 1		Model 2		Model 3	
		*b*	*t*	*b*	*t*	*b*	*t*
Step 1	ESH	0.551	14.02***	0.91	3.856***	−0.39	−0.415
	ER-R	1.146	12.40***	3.53	6.147***	2.25	1.764
	ER-S	−1.264	13.89***	−2.18	−4.060***	−4.44	−3.513***
	DIUT	−0.573	−4.24***	0.78	0.838	4.35	1.212
	Gender	−4.499	−4.21***	−4.91	−4.269***	−4.59	−3.903***
Step 2	ESH*ER-R			−0.011	−1.808	0.01	0.299
	ESH*ER-S			0.01	1.210	0.04	2.319*
	ESH*DIUT			−0.01	−1.328	−0.09	−2.058*
	ER-R*ER-S			−0.01	−2.961**	0.02	1.619
	ER-R*DIUT			−0.13	−6.122***	−0.28	−2.356*
	ER-S*DIUT			0.13	6.270***	0.20	1.898
Step 3	ESH*ER-R*ER-S					−4.34	−2.411*
	ESH*ER-R*DIUT					0.002	1.398
	ESH*ER-S*DIUT					−7.91	−0.557
	ER-R*ER-S*DIUT					−3.09	−0.575
	R^2^	0.749		0.779		0.787	
	ΔR^2^	0.561		0.607		0.619	

df = 97 for model 3; b = unstandardized coefficients

*p < 0.05, **p < 0.01, ***p < 0.001

The assumptions underlying the regression model were subjected to a comprehensive assessment, and it was determined that they were met. The linearity of the model was validated through the examination of partial regression plots and a plot of studentized residuals against predicted values. The independence of the residuals was indicated by the value of the Durban-Watson statistic, which was 1.22. Homoscedasticity was identified upon visual examination of a plot of studentized residuals against unstandardized predicted values. The issue of multicollinearity was not present, as indicated by the tolerance values exceeding the value of 0.1. No studentized deleted residuals were observed to exceed ± 3 standard deviations, and no leverage values were found to exceed 0.2. Additionally, Cook's distances remained below 1, thereby verifying the assumption of normality through a Q–Q plot (Model 3).

**Fig. 1:**
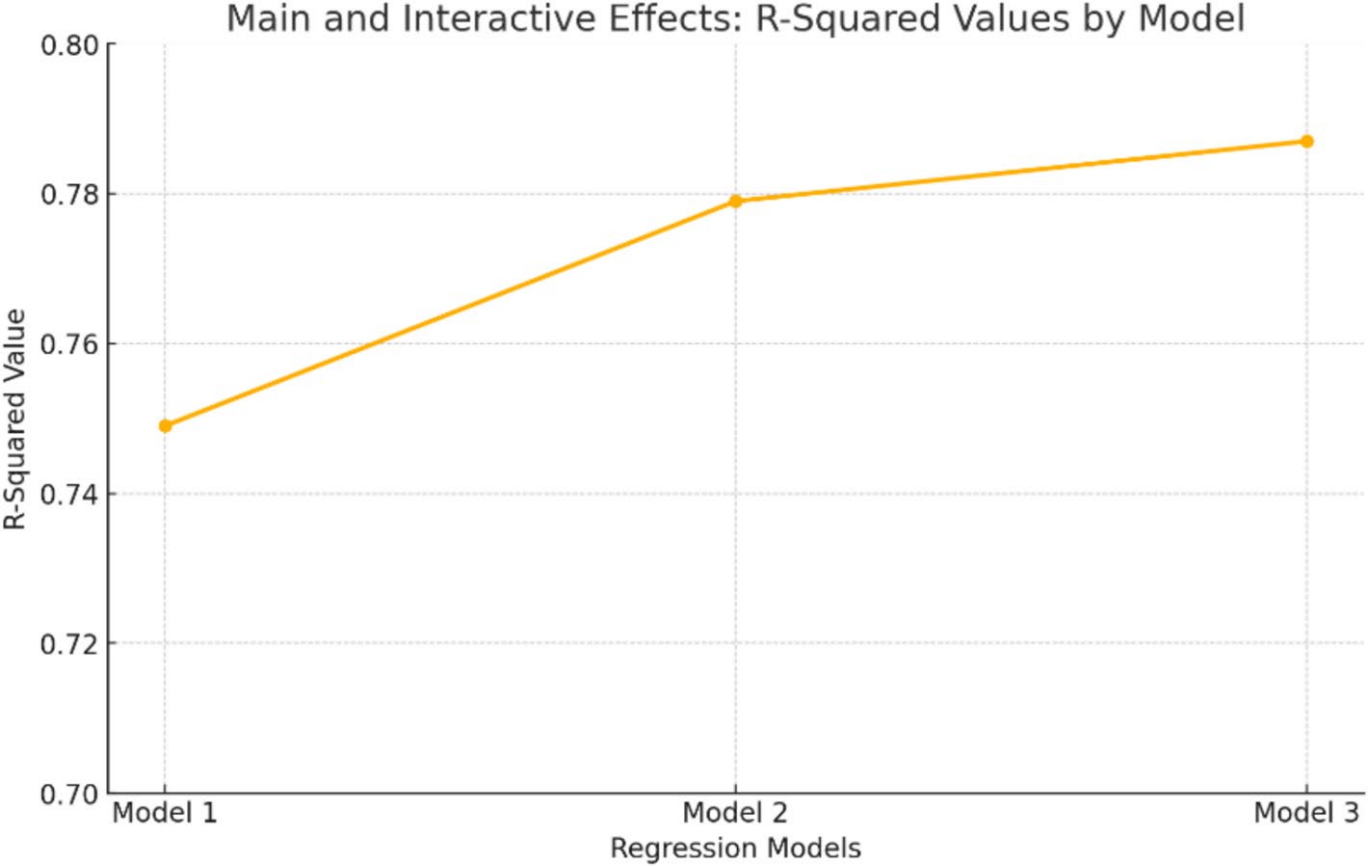
R-Squared values across regression models for predicting internet addiction

As demonstrated in Fig. 1, the R-squared value increases across models, thus indicating the predictive power of the variables.

**Fig. 2:**
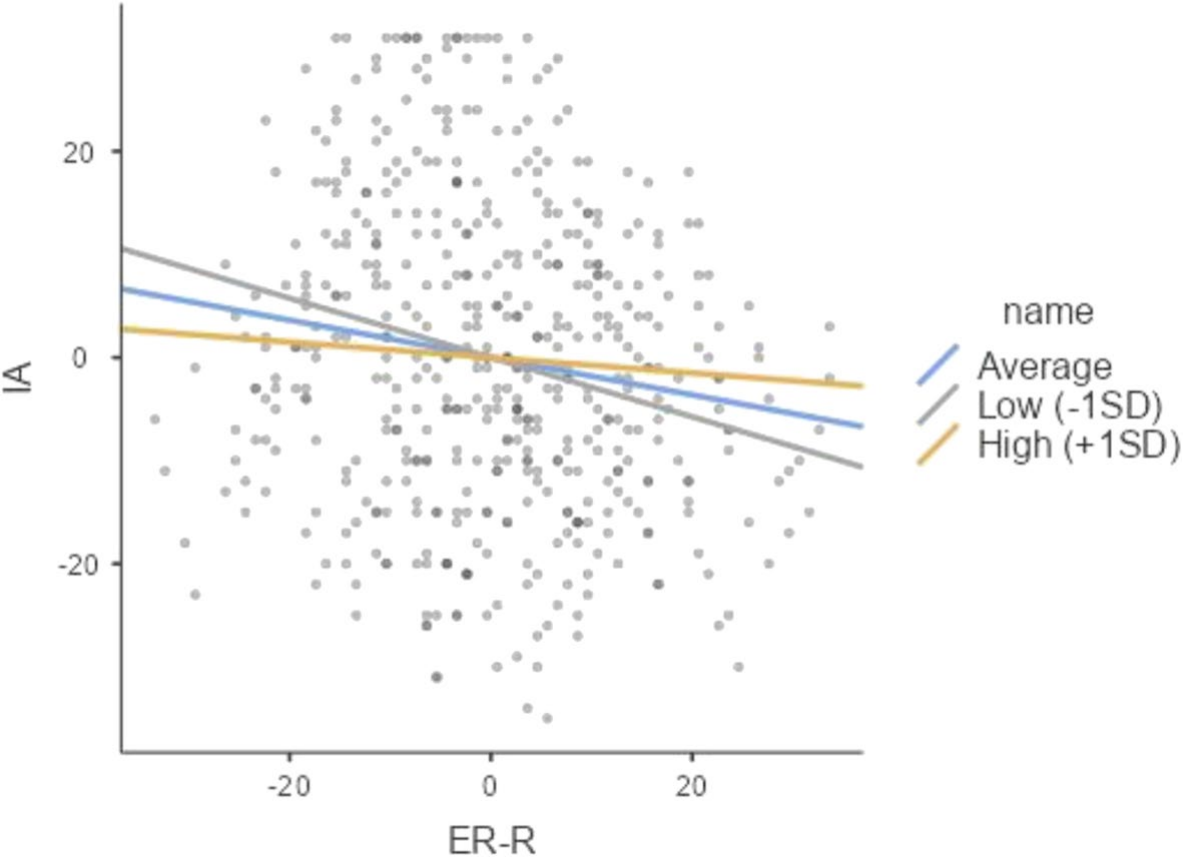
A straightforward examination of the correlation between internet addiction to ER-R, stratified by DIUT

A statistically significant full model was constructed to predict IA (Model 3), which incorporated variables for gender, DIUT, ESH, and ER skills (reappraisal and suppression). The model demonstrated an R2 of 0.787, F(25,466) = 33.2, and a p-value of less than 0.001, indicating a strong correlation between the model and the observed data. Additionally, the adjusted R2 was 0.621, further supporting the model's predictive power. The incorporation of ESH (Model 2) resulted in a statistically significant enhancement in the R^2^ value of 0.054, F(10,476) = 6.66, with a p-value less than 0.001. Furthermore, the subsequent incorporation of ER skills (reappraisal and suppression) in Model 3 yielded an additional increase of R^2^ by 0.026 (F(10,466) = 3.33, *p* < 0.001), indicating a substantial contribution of both ER and ESH skills to the prediction of IA, above and beyond the influence of demographic factors (Model 3 and Model 1) as demonstrated in Fig. 2.

Post hoc testing revealed that ER-R was a negative predictor of internet addiction among low and average DIUT participants (simple slopes = −0.2876 and −0.1813, ts = −3.97 and −3.52, ps < 0.001). Nevertheless, no significant correlation was observed between ER-R and internet addiction (simple slope = −0.0750, t = −1.08, ns) among participants with high DIUT (see Fig. 1). The findings show that ER-R level has a protective effect against internet addiction.

## Discussion and Conclusion

This study aimed at investigating whether ESH and ER can predict internet addiction and how they interact. Gender and DIUT were also examined as possible moderators of the relationship. The results indicate that IA, ESH, ER and DIUT are only slightly above the moderate level in PSTs. In the process of digital transformation, the IA levels are seen as the result of the idea that the faculty must take an active role in the pursuit of technology, which is the case in models like TPACK [102], SAMR [112], and digital competency frameworks (DigCompEdu) [114]. Similarly, as suggested by models such as the self-regulated learning model [164], motivation and self-determination theory (SDT) [118] in relation to ESH, the need to be successful in public personnel selection exam to be appointed to state institutions as a teacher known as the KPSS in Turkey, which PSTs must pass in order to be appointed to the job in Turkey, comes to the forefront, as does the need to move outside of school lessons and curriculum while preparing for the examination. The need for PSTs to have a high level of emotional intelligence comes to the forefront from the perspective of the cognitive behavioral theory (CBT), emotion regulation theory (ERT), and emotion management theory (EMT) models of the content provided in PST education. This need leads to teacher training programs emphasizing the importance of emotional regulation.

Our findings are consistent with Gross and John's [68] theory of emotional regulation. In accordance with the tenets of the emotion regulation theory [66], our findings revealed that DIUT, when considered in conjunction with ER-R, was a significant predictor of Internet Addiction among preservice teachers. Specifically, our data indicated that PSTs exhibiting high levels of DIUT (+ 1 SD) exhibited relatively low levels of Internet Addiction, a trend that was independent of ER-R. Conversely, our results demonstrated that higher levels of ER-R were associated with lower levels of Internet Addiction, particularly among those PSTs exhibiting average levels of DIUT, as opposed to those exhibiting low levels of DIUT. DIUT can be seen as a risk factor [23, 41, 91, 130, 156]. In other words, high DIUT, due to its association with decreased cognitive functioning – inhibition, decision-making, using working memory, learning, problem solving, logical thinking, calculation, literacy- [22, 44, 123, 161, 162], physical [133], social [88] and academic performance [85], may harden optimal engagement with internet and may result in diversion to task-irrelevant work and thus the neglect of tasks with an approaching deadline. Therefore, limiting the extent of internet use to that required for the task at hand may help to prevent the development of addictive behavior patterns associated with excessive internet use. Conversely, preservice teachers exhibiting typical levels of ER-R are likely to have developed effective coping strategies for disengagement, thereby reducing the probability of maladaptive behaviors when confronted with high degrees of DIUT.

The findings of this study align with those reported by van Deursen et al. [146], Tang et al. [138], Brand et al. [16], and Caplan [25]. Previous studies have demonstrated that internet use disorder reactivity acts as a risk factor that modulates the impact of externalizing regulatory problems (ER-R) on internalizing problems. However, the present study provides the first evidence that ER-R can act as a buffer against internet addiction in contexts of DIUT, to the best of our knowledge.

In addition, a four-way interaction was observed between gender, DIUT, ER, and ESH. Among PSTs, a negative association was observed between internet addiction and ER-R among participants with medium and low DIUT levels. The aforementioned interaction model also provided support for the risk-protective model, indicating that elevated DIUT may exert a detrimental impact on internet addiction in the context of ESH [156]. This finding is in alignment with evidence presented in previous research, indicating that heightened DIUT may serve as a protective mechanism, attenuating the potentially detrimental consequences of negative ER-S on an individual's psychological adjustment [39, 68, 91].

Additionally, the results demonstrated a significant main effect of ESH in predicting internet addiction levels in a positive way, as evidenced by the regression and correlational analyses. This relationship aligns with that posited by earlier studies which suggest that adverse emotional reactions (such as disengagement from extracurricular activities) may render individuals vulnerable to the development of maladaptive behavioral tendencies, including the problematic use of the internet (e.g., [91, 156]).

Several potential explanations for these findings have been put forth. First and foremost, it is plausible to suggest that PSTs' perceptions of ESH may be an integral component in elucidating the underlying factors that shape PSTs' study habits, negative affect, and lack of emotion regulation [116]. Consequently, those inclined to engage in risky behaviour may also display symptoms associated with internet addiction and other forms of maladjustment (see, for instance, [85, 145]). It is likewise conceivable that PSTs exhibiting comparatively diminished ESH may have been subject to less encouraging or supportive facilitation by lecturers [10, 86].

Should lecturers fail to enhance the ESH of PSTs, it may be reasonably presumed that they will be unable to provide the level of care and supervision that is appropriate for the students in question, as evidenced by the research of both Astin (1999) and Bray [17]. As a result of insufficient attention from their tutors, those training to become teachers may be susceptible to developing an addiction to the internet. Those in pre-service teacher (PST) training who experience a lack of academic discussion at residential institutions may view virtual exploration, interaction, and communication on the internet as an escape from such uninspiring environments. Additionally, they may perceive the internet as a valuable source of support [7, 75] and a place to feel positive identity and self-efficacy as a learner and human being.

It is important to consider the limitations of this study when interpreting the results. Firstly, it should be noted that ESH and ER were measured using self-report scales, which may have introduced a degree of bias into the findings. This is because participants may have responded in a socially desirable manner (see [47, 68]). It is recommended that subsequent studies incorporate observational or third-party assessments of ESH and ER. Secondly, the cross-sectional design employed in this study precludes the establishment of causal relationships. Longitudinal research would facilitate a greater understanding of the directionality of relationships between ESH, ER and internet addiction, and would permit a greater insight into how these factors change over time [71] such as significantly spiking or subsiding due to different personal factors and societal conditions. Thirdly, although the participants were instructed to complete the scales within a specified time frame, variations in timing and setting could have influenced their responses. A further limitation of the study was the lack of control over the data collection setting. It may be beneficial to collect data in a controlled manner to ensure more consistent outcomes.

A further limitation of the study is that the sample was drawn exclusively from pre-service teachers in education faculties. Consequently, the findings may not be applicable to other populations or contexts. It would increase the external validity of the study if it were to be replicated with a more diverse range of samples (see [60, 129]). It would also be interesting to compare such preservice teachers to inservice ones at different experience levels. Similarly, comparing teachers who only teach online to those in face-to-face educational settings would also be an interesting area to pursue; especially, given that teaching in online formats has become increasingly common and expected after the COVID-19 pandemic. Are those who have shifted to primarily teaching online displaying more negative internet addition traits than those who remain in physical education settings? Might they have acquired unique coping skills or support communities?

A fifth limitation is that the study did not examine additional potentially relevant factors, such as personality traits or academic stress levels, which could have influenced the findings. The inclusion of such additional variables could facilitate a more comprehensive understanding of the factors contributing to internet addiction [74, 87, 132]. Furthermore, the measures of internet addiction and ESH were based solely on self-report data, which may introduce shared method variance. It would be beneficial for future research to employ multiple measures or methods, such as parental or peer reports of ESH or clinical assessments of internet addiction, to enhance the validity of the findings.

Notwithstanding the aforementioned limitations, the present study also possesses a number of notable strengths. Firstly, the study includes a substantial and heterogeneous cohort of preservice educators from a multitude of academic institutions in Turkey. This enhances the generalizability of the findings across diverse educational contexts. Furthermore, the study adopts a distinctive approach in examining the interplay between extracurricular study habits (ESH) and emotion regulation skills (ER) in the prediction of internet addiction, thereby offering a more nuanced understanding of the collective influence of these factors on problematic internet use. A power analysis demonstrated that a sample size of a minimum of 150 was required to detect a moderate interaction effect. With a total of 492 participants included in the study, the sample size was sufficiently powered to reliably test such interactions. Additionally, the study utilized validated scales for ESH, ER, and internet addiction, ensuring the accurate capture of the relevant constructs. This methodological rigor contributed to the reliability and validity of the findings.

In conclusion, the current study extends the existing research by proposing preliminary evidence that extracurricular study habits (ESH) and emotion regulation skills (ER) serve as moderating variables in the relationship between internet addiction and individual characteristics among prospective educators. Our findings also indicate that elevated levels of ESH and ER-R, coupled with diminished levels of DIUT and ER-S, may serve as protective elements, thereby potentially mitigating the likelihood of internet addiction, particularly in instances where both competencies are cultivated. These findings reinforce the importance of examining individual resources, such as behavioral habits and emotional abilities, to gain insight into the contributing factors to internet addiction. In the end, this study emphasizes the significance of cultivating robust study habits and emotional regulation competencies as preventive measures against problematic internet usage among PSTs within academic contexts. In particular, the acquisition of emotional regulation skills by pre-service teachers should be made a part of the education process. It is vital now to explore and reveal the nuances and caveats involved in such cultivation.

## Data Availability

The data that support the findings of this study are available from the corresponding author, upon reasonable request.
